# Structural, Mechanical, and Electronic Properties of High-Hardness Silicon Tetranitride

**DOI:** 10.3390/molecules30224357

**Published:** 2025-11-11

**Authors:** Lulu Liu, Jiacheng Qi, Chi Ding, Dinghui Wang, Shoutao Zhang

**Affiliations:** 1 School of Electronic Engineering, Nanjing Xiaozhuang University, Nanjing 211171, China; 2 National Laboratory of Solid State Microstructures & Collaborative Innovation Center of Advanced Microstructures, School of Physics, Nanjing University, Nanjing 210093, China; 3 Jiangsu Physical Science Research Center, Nanjing 210093, China; 4 School of Materials Science and Physics, China University of Mining and Technology, Xuzhou 221116, China; 5 State Key Laboratory of Integrated Optoelectronics and Key Laboratory of UV-Emitting Materials and Technology of Ministry of Education, School of Physics, Northeast Normal University, Changchun 130024, China; zhangst966@nenu.edu.cn

**Keywords:** high-hardness materials, first-principles prediction, Si–N compounds, high-pressure, mechanical properties, high-energy-density materials

## Abstract

Materials with high hardness are critical for industrial and aerospace applications, prompting the search for novel compounds with robust covalent networks. Using a first-principles structure prediction method, we systematically explored the phase stability of Si–N compounds under high pressure. We identified two thermodynamically stable phases: Si_6_N with *P*-1 symmetry and SiN_4_ with space group *R*-3*c*. Phonon spectra and ab initio molecular dynamics simulations confirm the dynamical and thermal stability of *R*-3*c* SiN_4_ at ambient pressure and up to 2000 K. Notably, *R*-3*c* SiN_4_ exhibits exceptional mechanical properties with a Vickers hardness of 31 GPa, a bulk modulus of 259.53 GPa, and a Young’s modulus of 485.38 GPa. Furthermore, SiN_4_ possesses a high energy density (1.1 kJ·g^−1^) and outstanding detonation pressure and velocity (228 kbar, 7.11 km·s^−1^), both exceeding those of TNT, making it a potential high-energy-density materials. In addition, electronic structure analysis reveals SiN_4_ has a band gap of 2.5 eV, confirming its nonmetallic characteristics and strongly covalent nature. These findings provide theoretical guidance for the future synthesis of Si–N phases and establish a foundation for designing novel materials that combine high hardness with high-energy density performance.

## 1. Introduction

High-hardness materials combine superior hardness with excellent resistance to deformation, maintaining stable physical and chemical properties under extreme conditions such as high temperature and high pressure [1]. These features make them indispensable in advanced manufacturing, aerospace engineering, and defense applications [2,3]. Motivated by rising industrial and technological demands, researchers are devoted to exploring and developing novel multifunctional hard materials [4,5].

In recent years, experimental and theoretical research on hard materials has concentrated on compounds composed of light elements such as boron, carbon, nitrogen, and oxygen, owing to their ability to form strong covalent bonds that result in high hardness and superior resistance to deformation [6]. Among these, nitrides have been extensively studied due to their stable covalent bond structure (single bonds, 167 kJ·mol^−1^; double bonds, 419 kJ·mol^−1^) [7,8,9]. High-pressure synthesis has become a key strategy for preparing polymeric nitrogen, and various polymeric nitrogen phases have been experimentally prepared under high-pressure and high-temperature conditions [10]. However, the synthesis of pure nitrogen materials remains challenging due to the extremely high pressures (>110 GPa) required and the kinetic instability of elemental nitrogen phases. To overcome these limitations, the introduction of other elements under compression to synthesize stable nitrides at a relatively low pressure has been proven to be an effective method. The current goal is not only to obtain stable nitrides but also to achieve materials with high hardness.

Notably, the silicon-nitrogen compounds have attracted considerable attention because the Si–N bond enables the formation of covalent networks, while the incorporation of Si facilitates the stabilization and synthesis of diverse nitrides, thereby enhancing the potential for designing new high-hardness materials [11,12,13,14]. For example, theoretical investigations by Cui et al. have predicted several novel high-hardness phases of Si_3_N_4_ through first-principles calculations, namely *t*-Si_3_N_4_, *m*-Si_3_N_4_, and *o*-Si_3_N_4_ [15]. These phases exhibit lower energies under specific pressures than the conventional trigonal *α*-Si_3_N_4_ and hexagonal *β*-Si_3_N_4_, and possess higher Vickers hardness values [16]. Experimentally, two major Si–N stoichiometric materials, Si_3_N_4_ and SiN_2_, have been synthesized. Using synchrotron X-ray diffraction under high pressure, Qin et al. identified a new monoclinic phase of Si_3_N_4_, providing new insights into its high-pressure behavior [17]. In addition, *α*-Si_3_N_4_ (*P*3_1_*c*), *β*-Si_3_N_4_ (*P*6_3_/*m*), and *γ*-Si_3_N_4_ (*Fd*3/*m*) have also been reported. Weihrich et al. synthesized a pyrite-type SiN_2_ with a *Pa*-3 structure, which remains energetically stable above 15 GPa [18]. Niwa et al. further synthesized SiN_2_ (*Pa*-3) above 460 GPa and successfully recovered it at ambient conditions [19]. By contrast, Jurzick et al. synthesized SiN_2_ at 140 GPa [20]. Recently, dense Si_3_N_4_ ceramics were successfully fabricated via high pressure (5.5 GPa) and high temperature (900–1400 °C) [21]. Beyond these stoichiometries, recent studies have expanded the compositional and structural diversity of the Si–N system. Si-rich nitrides such as Si_64_N with cubic *P*-43*m* and rhombohedral *R*3*m* structures [22], as well as Cr-incorporated Si–N compounds forming bulk Cr_2_SiN_4_ phases [23] have been reported. However, these theoretical and experimental studies mainly focused on the nitrogen-rich Si_3_N_4_ and SiN_2_ compounds. In contrast, the existence of silicon-rich nitrides or other nitrogen-rich polymorphs in the Si–N system remains underexplored. Moreover, the potential existence of additional high-hardness structures in this system is still unknown. In addition, the systematic investigation of their mechanical and electronic properties is crucial for expanding the application potential of Si-N-based materials [24,25,26]. Sylvain Pitié et al. predicted several (meta)stable Si_x_N_γ_ phases, including SiN, Si_3_N_4_, SiN_2_, SiN_4_, SiN_6_, and SiN_20_, and identified nitrogen-rich Si–N compounds as high-energy-density materials [27]. Similarly, Paras Patel et al. predicted metastable Cc-SiN_6_ as a promising silicon-based high-energy-density material with high stability and strong detonation performance [28]. Other studies have also explored the Si–N system [29], with early work focusing mainly on nitrogen-rich silicon nitride phases. In contrast, the present study extends the investigation to more silicon-rich compositions and to properties that have remained largely unexplored in this system. Therefore, exploring new Si-N structures is not only critical for discovering new materials, but also provides reliable theoretical guidance for the experimental synthesis of multifunctional Si-N compounds in the future.

In this work, we employed an ab initio structure prediction method [30,31] to comprehensively explore the high-pressure phase stability and material properties of Si–N compounds, leading to the identification of several novel thermodynamically stable phases, including SiN_4_ and Si_6_N. Phase diagram analysis indicates that Si_6_N becomes stable at approximately 25 GPa, while SiN_4_ becomes stable at around 29 GPa. Notably, *R*-3*c* SiN_4_ remains kinetically, dynamically, and thermally stable under both ambient and high-pressure, high-temperature conditions. Mechanical properties analysis indicates its outstanding hardness and elasticity. Furthermore, explosive performance calculations suggest that *R*-3*c* SiN_4_ exhibits a high detonation pressure of 228 kbar and detonation velocity of 7.11 km·s^−1^, underscoring its potential as a multifunctional material.

## 2. Results and Discussion

### 2.1. Stable Si−N Compounds

**Figure 1:**
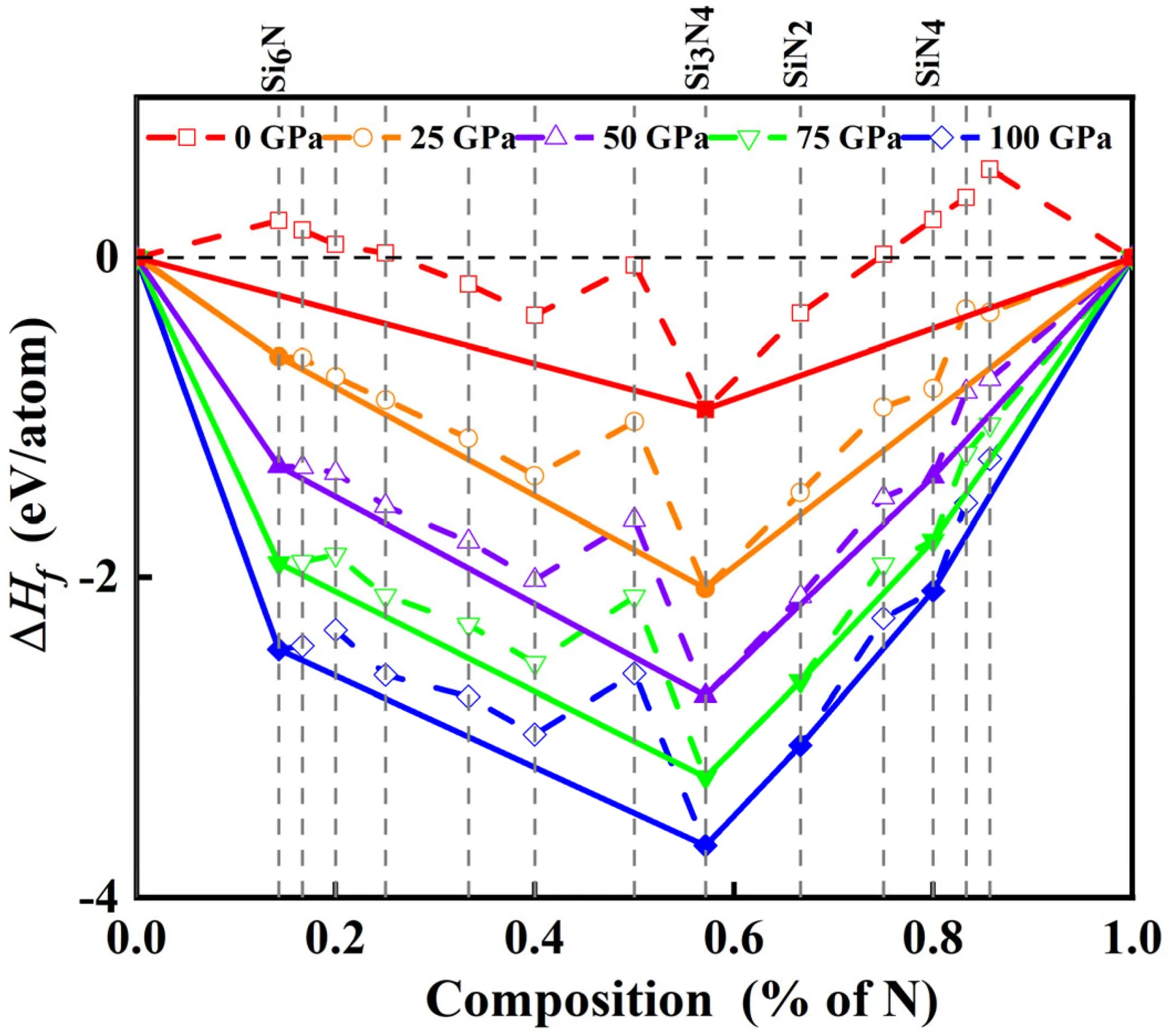
Calculated formation enthalpies (Δ*H_f_*) per atom of various Si–N compounds with respect to decomposition into solids Si and N_2_ at 0, 25, 50, 75, and 100 GPa, respectively.

To assess the thermodynamic stability of the predicted structures, the relative stability of Si_x_N_y_ (x = 1–6, y = 1; x = 1, y = 1–6; x = 3, y = 4) compounds across varying pressures (i.e., 0, 25, 50, 75, and 100 GPa) was systematically examined through convex hull diagrams, as shown in Figure 1. The formation enthalpy per atom was obtained using the relation:

(1) $$∆H_{f}\;=\;\frac{1}{\left(x+y\right)}[H({\mathrm{Si}}_{x}N_{y})-xH(\mathrm{Si})-\frac{y}{2}H\left(N_{2}\right)]$$

where *H*(Si_x_N_y_) is the enthalpy of Si_x_N_y_ compound, *H*(Si) and *H*(N_2_) are the enthalpies of elemental silicon and nitrogen phases. For reference, the diamond-structured Si was adopted. The definition of the nitrogen elemental reference states is indeed a critical issue in evaluating formation enthalpies under pressure, as elemental nitrogen undergoes a series of pressure-induced structural transformations and exists in different allotropic forms at different pressures. In the 0–100 GPa range, experimental studies indicate that, after the structural transition from *Pa*-3 to *P*4_1_2_1_2 molecular nitrogen, the phase can persist to pressures above 100 GPa because of the high kinetic barrier for further phase transitions. So, *Pa*-3 and *P*4_1_2_1_2 molecular nitrogen were used as reference phases for computing the formation enthalpy [32,33]. As shown in the convex hull diagrams (Figure 1), the formation enthalpy per atom is obtained by dividing the total formation enthalpy of Si_x_N_γ_ by the total number of atoms (x + y). Solid symbols connected by solid lines denote thermodynamically stable compositions. In contrast, hollow symbols linked by dashed lines correspond to metastable or unstable phases. The negative formation enthalpy of cubic Si_3_N_4_ (*Fd*-3*m*) confirms its stability under ambient conditions (~0 GPa), consistent with previous theoretical and experimental reports demonstrating successful synthesis in a diamond anvil cell. Likewise, the predicted *Pa*-3 SiN_2_ phase remains stable under high pressure, further validating the reliability of the employed structure-search methodology [20].

In addition to Si_3_N_4_ and SiN_2_, two additional compositions, SiN_4_ and Si_6_N, are predicted to be thermodynamically stable. Among them, Si_6_N is a newly identified composition. Although SiN_4_ has been reported previously [27], it was found to be metastable when evaluated against theoretical elemental reference states. By contrast, using experimentally established elemental reference phases, the present calculations indicate that SiN_4_ is thermodynamically stable. At ambient pressure, both lie slightly above the convex hull, indicating slightly metastable behavior. Upon compression, the formation enthalpy of Si_6_N decreases sharply, and this phase becomes thermodynamically stable above ~25 GPa, indicating that pressure effectively reduces the energetic barrier for forming this Si-rich compound. By contrast, SiN_4_ displays a more gradual enthalpy-pressure dependence, remaining metastable over intermediate pressures and only approaching the convex hull near 50 GPa. To determine the precise pressure, enthalpy difference calculations show that SiN_4_ is stabilized at ~3 GPa relative to elemental Si and N_2_, and at ~29 GPa relative to competing phases such as Si_3_N_4_ (see Supplemental Material, Figure S1). Overall, the enthalpy-pressure relationships indicate that the synthesis pressures for newly identified Si_6_N and SiN_4_ are below moderate pressure (~50 GPa). In contrast, nitrogen-rich compositions generally require higher stabilization pressures than Si-rich ones, reflecting the significant energetic barriers associated with breaking nitrogen’s triple bond and forming polymeric nitrogen frameworks. These results elucidate the pressure-driven stabilization pathways of both Si-rich and N-rich Si–N compounds and highlight potential routes for synthesizing novel structures.

### 2.2. Crystal Structures

**Figure 2:**
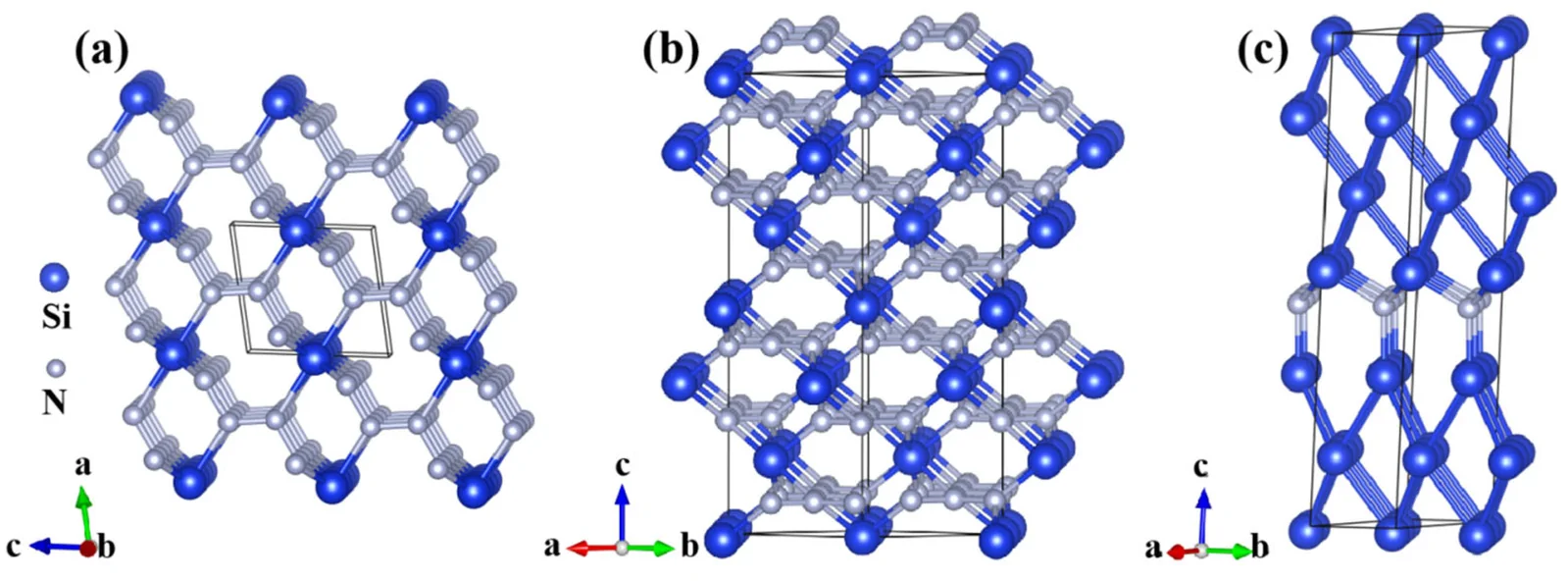
(**a**) *P*-1 SiN_4_ at 0 GPa. (**b**) *R*-3*c* SiN_4_ at 0 GPa. (**c**) *Cm* Si_6_N at 25 GPa. Si atoms are shown in blue, and N atoms in gray.

Two SiN_4_ polymorphs (*P*-1 SiN_4_ and *R*-3*c* SiN_4_) and the *Cm* Si_6_N phase are predicted to be thermodynamically stable at high pressure. The atomic configurations of the predicted phases are shown in Figure 2. As shown in Figure 2a, the *P*-1 SiN_4_ phase crystallizes in a triclinic symmetry, where each Si atom is coordinated by six N atoms to form a structural unit. Among the coordinated N atoms, four are shared with adjacent units, while the remaining two are connected to other units through additional N linkages. The average Si–N bond length is 1.89 Å, whereas all N–N bonds maintain a uniform length of 1.31 Å at 0 GPa. In contrast, the *R*-3*c* SiN_4_ phase belongs to the trigonal crystal system and is stabilized by adopting a three-dimensional octahedral network. At 0 GPa, all Si–N bonds are identical, with a length of 1.91 Å, while all N–N bond lengths are 1.32 Å. These bond lengths lie within the characteristic range of stable covalent bonds. Each Si atom is bonded to six surrounding N atoms, forming an octahedral coordination geometry. Meanwhile, the ∠NSiN angle is 90 degrees, maximizing the balance of atomic repulsion. This uniform octahedral coordination imparts high bond energy, ensuring robust interatomic cohesion and compact connectivity resistant to dissociation. As shown in Figure S2, the lattice constant (a = b = c) of the *R*-3*c* SiN_4_ decreases continuously with increasing pressure, exhibiting an overall contraction of approximately 10% at 100 GPa. Overall, the cooperative bonding interactions effectively constrain atomic displacement, thereby reinforcing the resilience of the crystal framework.

For the *Cm* Si_6_N phase, which crystallizes in a monoclinic symmetry, the average Si–N bond length is 1.79 Å. In addition, Si-Si bonds have an average length of 2.51 Å at 25 GPa. Although the relatively longer bond lengths suggest lower bond energy and slightly weaker interatomic interactions, the overall configuration represents a balanced bonding state that preserves structural stability. The Si–N bonds provide supplementary connectivity channels within the crystal lattice, further reinforcing its structural stability in three-dimensional space.

### 2.3. Mechanical, High-Energy Density, and Electronic Properties

A quantitative assessment of hardness is critical to the theoretical design of high-hardness materials. Several semi-empirical models correlate hardness with the intrinsic properties of crystals [34,35,36,37]. Although these models employ different calculation approaches, their predicted values are generally very similar [38,39]. Among these, the Chen model is widely used and has been successfully applied to predict the hardness of many single-crystal materials, showing good agreement with experimental data [39,40,41,42,43,44,45,46]. For instance, it accurately predicts the Vickers hardness of diamond and cubic boron nitride (*c*-BN) in close agreement with measured values. Its broad applicability and proven reliability make it well-suited for the comparative screening of high-hardness materials, and it was therefore adopted in this study. While the model was originally formulated for polycrystalline materials, it is applied here to single-crystal structures, disregarding the influence of plastic deformation and dislocation motion. Therefore, the obtained values correspond to the intrinsic hardness of the materials under idealized conditions. Our analysis reveals that *R*-3*c* SiN_4_ has a Vickers hardness of 31 GPa, thereby surpassing the empirical threshold of 30 GPa that defines high-hardness materials. For the other two structures, the Vickers hardness values of *P*-1 SiN_4_ and *Cm* Si_6_N phases are 17 and 8 GPa, respectively, both much lower than that of *R*-3*c* SiN_4_. Evaluating elastic constants is essential for understanding the mechanical properties of the predicted Si–N compounds. The calculated elastic constants show that all values of the *R*-3*c* SiN_4_ satisfy the established mechanical stability criteria [47]. In addition, we computed the bulk modulus (*B*), shear modulus (*G*), Young’s modulus (*E*), and Poisson’s ratio (*ν*) to further assess the mechanical properties (Table 1). The *R*-3*c* SiN_4_ phase displays an exceptionally high bulk modulus of 259.53 GPa, reflecting its outstanding resistance to volume compression. Meanwhile, the shear modulus and Young’s modulus reach 204.23 and 485.38 GPa, respectively, indicating superior resistance against shape deformation and external stress. Poisson’s ratio is 0.18. Materials with a low Poisson’s ratio have very little change in lateral dimensions when subjected to axial load. These results demonstrate that the *R*-3*c* SiN_4_ combines remarkable hardness with extraordinary incompressibility and shear strength, making it a promising high-hardness material. The mechanical moduli of the *P*-1 SiN_4_ are listed in Table 1; all of which are inferior to those of *R*-3*c* SiN_4_. A comprehensive comparison integrating key properties of the *R*-3*c* and *P*-1 SiN_4_ is presented in Table S1 in the Supplemental Material.

**Figure 3:**
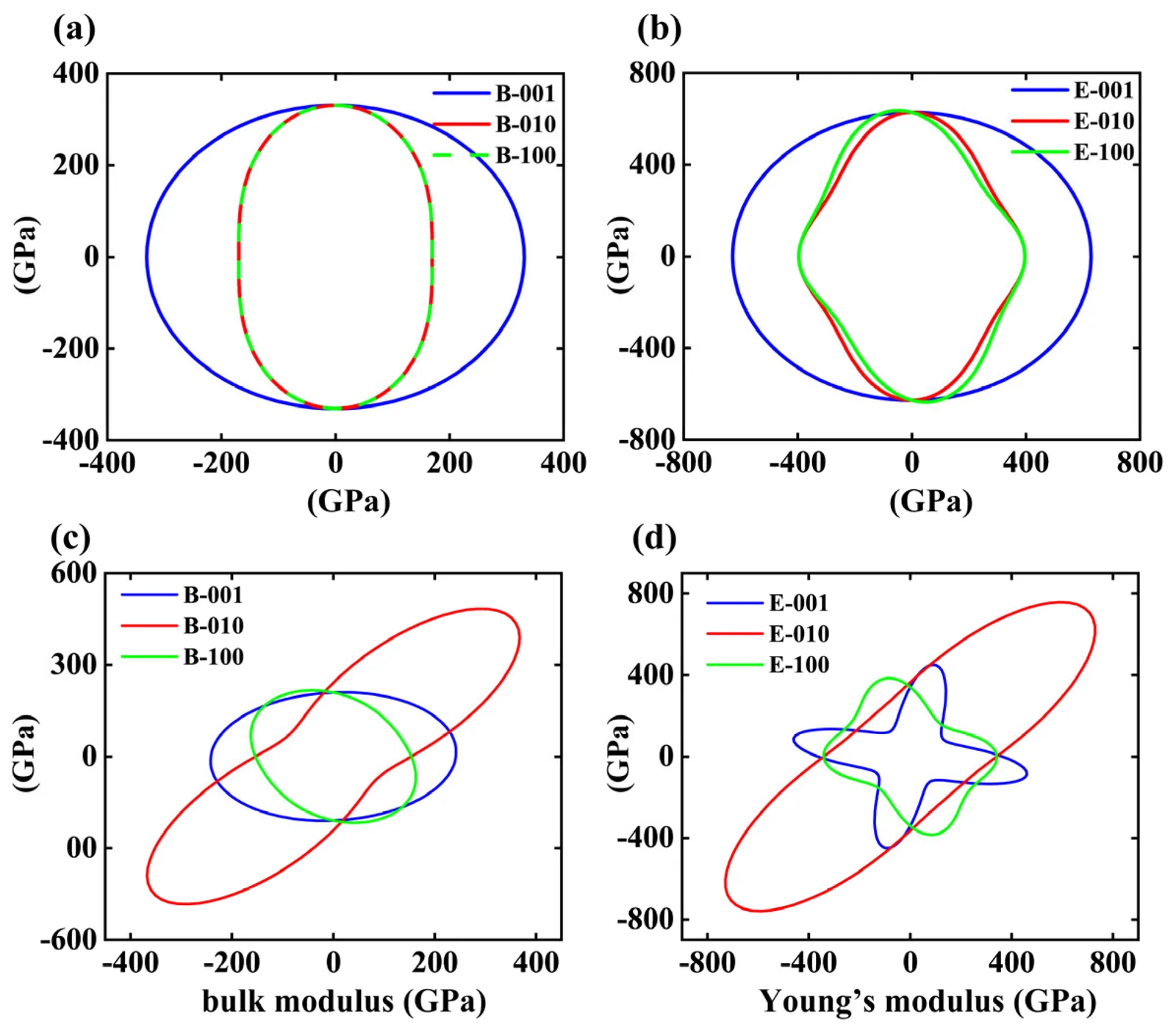
Projections of bulk modulus and Young’s modulus on selected crystallographic planes for the (**a**,**b**) *R*-3*c* SiN_4_ and (**c**,**d**) *P*-1 SiN_4_.

The bulk and Young’s moduli were analyzed in spherical coordinates, and the corresponding elastic anisotropy was visualized by projecting these moduli onto selected crystallographic planes (001), (010), and (100) based on the formulations. The results for these two SiN_4_ polymorphs are illustrated in Figure 3. For the trigonal *R*-3*c* SiN_4_ phase, the contour projections of the bulk modulus on the (010) and (100) planes nearly overlap. This feature indicates that the bulk modulus is essentially isotropic in these directions. The (001) plane exhibits an almost spherical distribution with slightly higher values than those on the (010) and (100) planes. A similar trend is observed for Young’s modulus on the (001), which exhibits an isotropic distribution analogous to that of the bulk modulus and is approximately twice the magnitude of the latter. In contrast, Young’s modulus on the (010) and (100) planes deviates from a perfectly spherical pattern, displaying only weak isotropy along these directions. These results indicate that the *R*-3*c* SiN_4_ structure possesses excellent incompressibility and shear resistance along all three crystallographic orientations. In contrast, the triclinic *P*-1 SiN_4_ phase exhibits pronounced elastic anisotropy due to its very low crystal symmetry. Both the bulk and Young’s moduli reveal significant directional dependence across all three planes, with the (010) plane showing the most distinct anisotropy, characterized by a rod-like contour. The (001) and (100) planes also display anisotropy, though to a lesser degree. Overall, a direct comparison between the two polymorphs highlights that the elastic anisotropy of both bulk and Young’s moduli is much more pronounced in the *P*-1 phase than in the *R*-3*c* phase. Furthermore, the lower moduli values in the *P*-1 structure are consistent with its reduced hardness. These observations indicate that even at the same stoichiometric ratio, variations in atomic bonding configurations can induce substantial differences in the mechanical behavior of the structures. Although the *Cm* Si_6_N phase satisfies the mechanical stability criteria, its moderate hardness of 8.8 GPa limits its practical relevance as a hardness material; therefore, we will not discuss this structure in detail in the subsequent description of properties. Considering its high hardness, large elastic moduli, and distinctive anisotropic features, the *R*-3*c* SiN_4_ phase emerges as a promising high-hardness material with superior mechanical performance and was thus selected as the primary candidate for subsequent in-depth analyses.

As can be seen from Figure 1, *R*-3*c* SiN_4_ has a positive formation enthalpy, which is about 0.238 eV·atom^−1^ higher than the enthalpy of elemental Si plus nitrogen at 0 K and 0 GPa. After detonation, *R*-3*c* SiN_4_ may decompose in the following way: SiN_4_ (solid) → Si (solid) + 2N_2_ (gas). Further calculations show that *R*-3*c* SiN_4_ can release about 1.1 kJ·g^−1^ of energy, reaching the standard of high-energy-density materials (1.0 kJ·g^−1^). Typically, detonation velocities (*V_d_*) and pressures (*P_d_*) provide insights into the material’s explosive power and performance, which can be estimated based on Kamlet–Jacobs empirical equation. This method has been successfully applied to evaluate the explosive properties of nitrides and other energetic materials [48,49,50,51,52]. The corresponding expressions are given below:

*V_d_* = 1.01(*N M*^0.5^ *E_d_*^0.5^)^0.5^(1 + 1.30 ρ) (2)

*P_d_* = 1.58 ρ^2^ *N M* 0.5 *E_d_*^0.5^ (3)

Herein, *N*, *M*, and ρ stand for the concentration of N_2_ in terms of moles per gram of explosive material (expressed as mol·g^−1^), the molecular weight of N_2_ gas (equivalent to 28 g·mol^−1^), and the density (measured in g·cm^−3^), respectively. In the above formulas, the units of gravimetric chemical energy density (*E_d_*) should be converted to kJ·kg^−1^. In addition, our research results show that *R*-3*c* SiN_4_ releases a large amount of nitrogen upon detonation, with a detonation pressure of 228 kbar and a detonation velocity of 7.11 km·s^−1^—both of which exceed those of the famous explosive material trinitrotoluene (TNT, 190 kbar for detonation pressure and 6.90 km·s^−1^ for detonation velocity) (Table S3) [53,54]. These data demonstrate that *R*-3*c* SiN_4_ may be a potentially high-energy-density material that exhibits excellent detonation performance.

**Figure 4:**
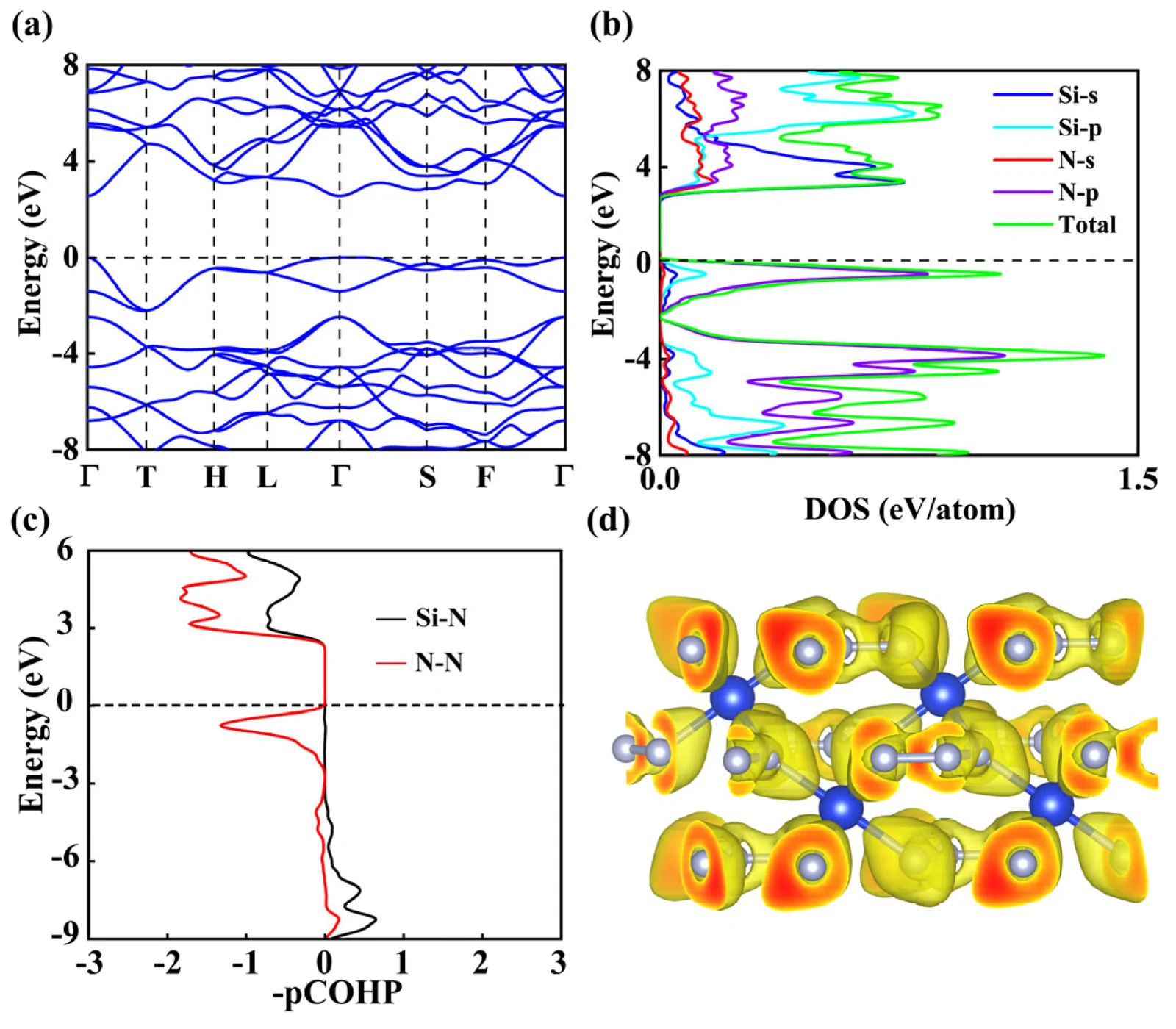
(**a**) Electronic band structure, (**b**) density of states (DOS), (**c**) minus projected crystal orbital Hamilton populations, and (**d**) three-dimensional planes of electron localization function of *R*-3*c* SiN_4_ at 0 GPa.

To systematically investigate the electronic properties and bonding characteristics of *R*-3*c* SiN_4_, we calculated its electronic band structure and density of states (DOS) at ambient pressure, as shown in Figure 4a,b. The band structure reveals a band gap of 2.5 eV, confirming the non-metallic nature of *R*-3*c* SiN_4_. Further band calculations at 100 GPa show that this structure has a band gap of 2.6 eV, which is almost the same as that at normal pressure, and still maintains non-metallic properties (Figure S3). The analysis of the total and partial DOS indicates that states near the Fermi level are primarily derived from the Si 3s and N 2p orbitals. The Si 3s states exhibit a prominent peak below the Fermi level, while the N 2p orbitals contribute significantly across a similar energy range, indicating strong s-p orbital hybridization. The hybridized orbitals suggest the formation of covalent bonds between Si and N atoms. For comparison, we also examined the *P*-1 SiN_4_ phase, which shows a semiconducting nature with a narrower band gap of 1.4 eV compared to that of *R*-3*c* SiN_4_ (see Figure S4 in the Supplemental Material).

To further clarify bonding and antibonding interactions, we employed the negative projected Crystal Orbital Hamiltonian population (−pCOHP), a widely used quantum chemical method. As shown in Figure 4c, a significant −pCOHP value appears below the Fermi level, confirming the existence of bonding states. Integral COHP (ICOHP) analysis of the *R*-3*c* SiN_4_ phase shows that the strength of the nearest-neighbor N–N bond (−14.13 eV per pair) is approximately twice that of the Si–N bond (−5.59 eV·pair^−1^). Similarly, the Integrated Crystal Orbital Overlap Population (ICOOP) values (0.40 eV per pair for N–N and 0.24 eV·pair^−1^ for Si–N) indicate that the N–N bond has greater orbital overlap and stronger covalency. The total Madelung (Ewald) energies per atom calculated using Mulliken and Rödinger charges are −4.10 eV and −3.09 eV, respectively, indicating that the contribution of ionic bonds to overall bonding is not negligible. These results indicate that N-N bonds in *R*-3*c* SiN_4_ dominate covalent interactions, while Si-N bonds play a relatively minor role, leading to a synergistic stabilizing effect of both covalent and ionic components. Covalent bonding behavior was also observed in the −pCOHP diagram of the *P*-1 SiN_4_ phase (Figure S5). For the *P*-1 SiN_4_ phase, the ICOHP value of the nearest-neighbor N-N bond is −14.83 eV·pair^−1^, approximately twice that of the Si-N bond (−5.96 eV·pair^−1^), with corresponding ICOOP values of 0.39 and 0.18 eV·pair^−1^, respectively. The calculated Madelung energies per atom are −4.18 eV (Mulliken) and −2.67 eV (Rödinger), which are nearly identical to those of the *R*-3*c* SiN_4_ phase. As shown in Table S1, the results suggest that these two phases have similar bonding characteristics, primarily composed of N-N covalent interactions and ionic stabilizing effects.

Further insight into the intrinsic bonding of *R*-3*c* SiN_4_ was obtained from the ELF (Figure 4d) and Bader charge analysis. The ELF exhibits strong localization around N atoms and along Si–N bonds, confirming the directional covalent nature of these interactions. The pronounced red regions near N highlight the concentration of electron density in these areas. Bader charge analysis showed that each Si atom donates approximately 3e, while only partial N atoms gain about 1e. This is not a complete transfer but rather a covalent sharing of electron density between Si and N atoms, primarily through covalent bonds. This charge redistribution stems from the strong electronegativity of nitrogen atoms, resulting in a shift in electron density toward neighboring N atoms. The Bader charge is consistent with the ELF results and the octahedral network observed in the *R*-3*c* SiN_4_ structure. These results provide consistent evidence for covalent bonding with significant ionic characteristics in *R*-3*c* SiN_4_. We also calculated the Bader charge of *P*-1 SiN_4_, which exhibits similar electron transfer characteristics to *R*-3*c* SiN_4_ as shown in Table S2.

### 2.4. Dynamic and Thermal Stabilities

**Figure 5:**
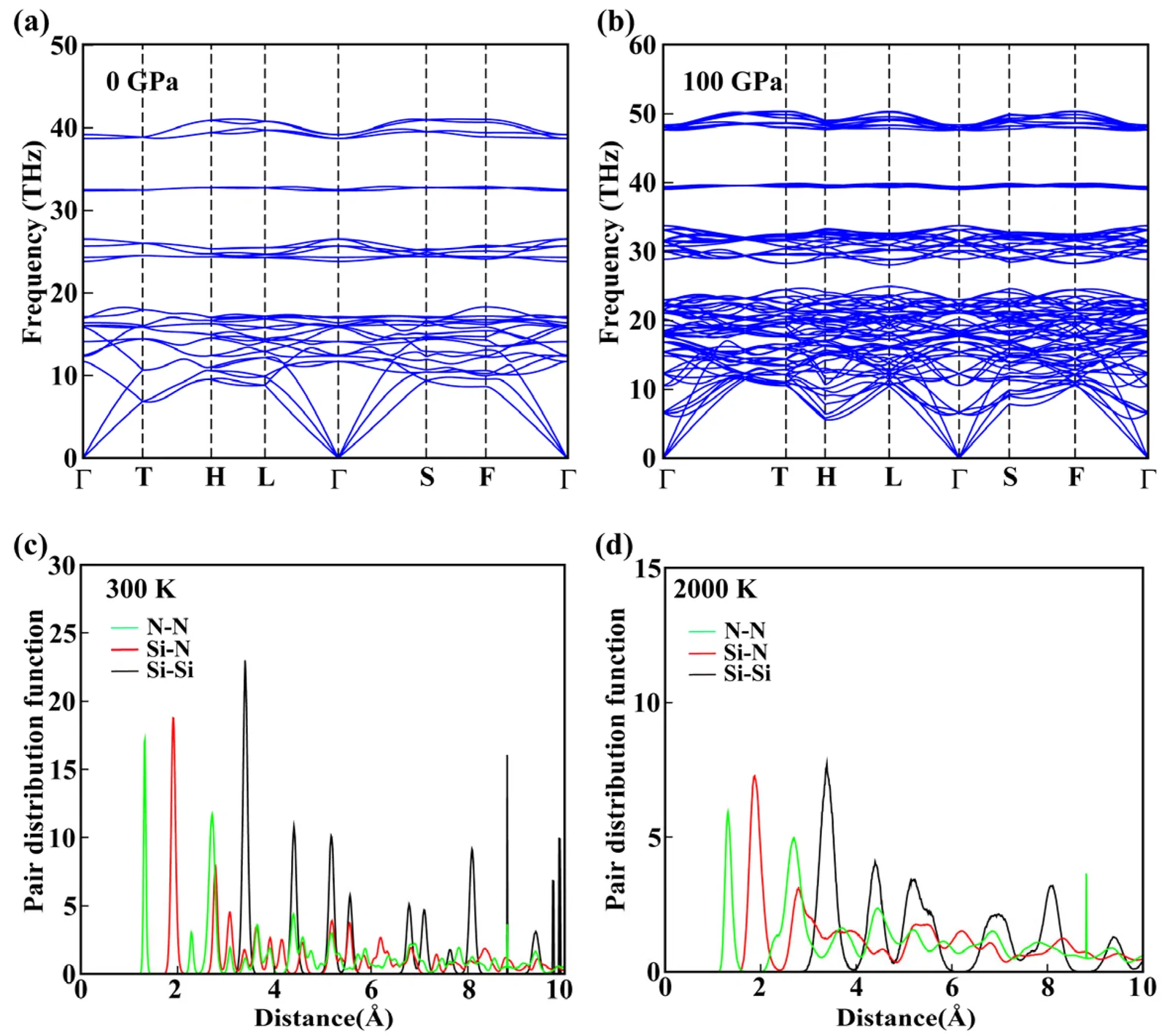
Dynamical and thermal stability analysis of *R*-3*c* SiN_4_. Phonon spectra at (**a**) 0 and (**b**) 100 GPa, respectively. Pair distribution functions at (**c**) 300 and (**d**) 2000 K, respectively.

To assess the dynamic stability of *R*-3*c* SiN_4_, we calculated its phonon dispersion curves at 0 and 100 GPa, as shown in Figure 5a,b. The phonon spectra exhibit no imaginary frequencies along the entire Brillouin zone, confirming the dynamic stability of *R*-3*c* SiN_4_ under both ambient and high-pressure conditions. Comparison of the phonon patterns reveals that increasing the pressure to 100 GPa shifts all vibration modes to higher frequencies, with a notable enhancement in the intensity of N atom vibrations in the middle and high-frequency regions. This behavior arises from the shortened interatomic distances and increased lattice stiffness under high pressure, which elevates the restoring force constants of atomic vibrations and suppresses the lattice vibrational degrees of freedom. We further computed the phonon spectra of the *P*-1 SiN_4_ phase, which confirms its dynamic stability at 0 GPa (Figure S6).

The thermal stability of *R*-3*c* SiN_4_ was evaluated using ab initio molecular dynamics (AIMD) simulations at 300 and 2000 K, as shown in Figure 5c,d. Equilibrium structures were extracted from the final steps of the AIMD runs. At 300 K, the bond length distribution for the shortest N–N bond (green line) is 1.32 Å, that for the nearest Si–N bond (red line) is 1.91 Å, and that for the Si–Si distance (black line) is 3.40 Å, which closely match those of the relaxed structure, indicating negligible deviation from the original bond lengths and confirming the stability of *R*-3*c* SiN_4_ at ambient temperature. At 2000 K, similar behavior is observed: the first peaks of all bond length distributions are aligned with those of the initial structure, demonstrating that the system preserves its bonding network under high-temperature conditions. As shown in Figure S7, the final structures obtained from AIMD simulations at both temperatures maintain the original bonding framework without significant deformation or distortion, confirming that *R*-3*c* SiN_4_ remains solid and structurally intact up to 2000 K. Pair distribution functions of *P*-1 SiN_4_ at 0 GPa and at 300 and 1000 K are presented in Figure S8. These results indicate excellent thermal stability over a wide temperature range, providing a solid theoretical basis for its potential experimental synthesis and high-temperature applications.

## 3. Calculation Methods and Details

The search for possible crystal structures was performed using the swarm-intelligence-based evolutionary algorithm as implemented in the CALYPSO code [30,31]. First-principles density functional theory (DFT) calculations [55] were employed to investigate the structural, electronic, elastic, and vibrational properties under high pressure, as well as to evaluate phase stability via total energy calculations. In recent years, DFT calculations, combined with the CALYPSO particle swarm optimization algorithm, have enabled the identification of thermodynamically stable structures given the chemical composition alone. These approaches provide predictive insights into phase stability and serve as a guide for experimental discovery of novel high-pressure materials [9,45,56,57,58,59]. Subsequent first-principles calculations, including structural optimization and electronic structure analyses, were carried out with the Perdew–Burke–Ernzerhof (PBE) [60] functional of the generalized gradient approximation (GGA) [61] as implemented in the Vienna Ab initio Simulation Package (VASP) [62]. The projector augmented wave (PAW) formalism [63] was employed with pseudopotentials explicitly considering the valence electrons, namely 3s^2^3p^2^ for Si and 2s^2^2p^3^ for N. Numerical accuracy was ensured by adopting a plane-wave cutoff energy of 700 eV and a Monkhorst–Pack *k*-point mesh with a reciprocal space resolution of 2π × 0.025 Å^−1^, resulting in a total energy convergence better than 1 meV per atom [64]. The elastic moduli were computed using the Voigt–Reuss–Hill averaging method [65,66]. The detonation velocity and pressure were estimated based on the Kamlet-Jacobs Empirical Equation [53,54]. Furthermore, the Vickers hardness was estimated using a microscopic hardness model [36]. The phonon dispersions were calculated using the direct supercell method implemented in the PHONOPY package (V.2.43.6) [67] in conjunction with the VASP code. For the *R*-3*c* SiN_4_ phase, 80-atom and 100-atom supercells were employed at 0 and 100 GPa, respectively. Ab initio molecular dynamics (AIMD) simulations were performed within the *NpT* ensemble employing a Langevin thermostat, based on the *R*-3*c* SiN_4_ supercell model containing 80 atoms [68]. A time step of 1 fs and a total simulation time of 8 ps were used in the molecular dynamics simulations. The electron localization function (ELF) was employed to analyze the electron localization behavior [69], and the results were displayed using the Visualization for Electronic and Structural Analysis (VESTA) software (Version 3) [70]. Chemical bonding characteristics were quantitatively analyzed through Crystal Orbital Hamilton population (COHP), Crystal Orbital Overlap Population (COOP), and Madelung energy calculations performed using the LOBSTER code with the PBEVaspFit2015 basis set [71]. The following basis functions were applied: Si: 3s, 3p and N: 2s, 2p. All calculations employed wavefunctions obtained from VASP using the PBE exchange–correlation functional and the PAW method. Additional computational details are provided in the Supplemental Material.

## 4. Conclusions

Using first-principles structure prediction methods, we systematically explored the high-pressure phase stability of Si–N compounds, identifying novel thermodynamically stable phases, including SiN_4_ and Si_6_N. Phase diagram analysis shows that Si_6_N stabilizes at ~25 GPa, while SiN_4_ becomes stable at ~29 GPa, indicating that their synthesis pressures are relatively low. Notably, SiN_4_ with *R*-3*c* symmetry remains kinetically and dynamically stable at both ambient and high pressures, and dynamically stable up to 2000 K. Mechanical evaluations reveal exceptional hardness (31 GPa) and high bulk (259.53 GPa), shear (204.23 GPa), and Young’s modulus (485.38 GPa). Its excellent explosive properties make this nitrogen-rich compound a potential high-energy-density material. Electronic structure analysis reveals a band gap of 2.5 eV, characterized by strong covalent bonds. The results demonstrate that *R*-3*c* SiN_4_ is a high-hardness material that combines high stability, mechanical strength, and high energy density. This work deepens our understanding of the structural, mechanical, and electronic properties of Si–N compounds and provides theoretical guidance for the synthesis of multifunctional nitride materials.

## Figures and Tables

**Table 1:** Calculated mechanical moduli and Vickers hardness values of *R*-3*c* SiN_4_ and *P*-1 SiN_4_. Except for ***v***, all other parameters are given in GPa.

	*B*	*G*	*E*	*v*	*H_V_*
*R*-3*c* SiN_4_	259.53	204.23	485.38	0.18	31
*P*-1 SiN_4_	227.38	139.45	347.35	0.25	17

## Data Availability

The original contributions presented in this study are included in the article/Supplementary Material. Further inquiries can be directed to the corresponding author.

## Supplementary Materials

The following supporting information can be downloaded at: https://www.mdpi.com/article/10.3390/molecules30224357/s1, Figure S1. Calculated enthalpy differences of *R*-3*c* SiN_4_ relative to the mixtures of Si_3_N_4_, Si, and pure nitrogen. Figure S2. Variation of the lattice constants (a = b = c) of *R*-3*c* SiN_4_ the unit cell with pressure. Figure S3. (a) Electronic band structure *R*-3*c* SiN_4_ at 100 GPa. Figure S4. (a) Electronic band structure and (b) dos of *P*-1 SiN_4_ at 0 GPa. Figure S5. The -PCOHP of *P*-1 SiN_4_ at 0 GPa. Figure S6. Phonon spectra of *P*-1 SiN_4_ at 0 GPa. Figure S7. (a) The structure after initial optimization, (b) final structure at 300 K, and (c) final structure at 1000 K of *R*-3*c* SiN_4_. Figure S8. Pair distribution functions of *P*-1 SiN_4_ at 0 GPa at 300 K (a) and 1000 K (b), respectively. Table S1. Comparison of mechanical, electronic, and bonding properties of *R*-3*c* and *P*-1 SiN_4_ phases. Table S2. Charge transfer in *P*-1 SiN_4_ at 0 pressure. Table S3. Calculated energy density (*E_d_,* kJ·g^−1^), detonation pressure (*P_d_,* kbar), and detonation velocity (*V_d_*, km·s^−1^) of *R*-3*c* SiN_4_. For comparison, the table also lists the explosion parameters of our calculated TNT and experimental TNT, where the superscript *expt* represents the experimental data.
