# Correction: Liu et al. Structural, Mechanical, and Electronic Properties of High-Hardness Silicon Tetranitride. *Molecules* 2025, *30*, 4357

**DOI:** 10.3390/molecules31162840

**Published:** 2026-08-14

**Authors:** Lulu Liu, Jiacheng Qi, Chi Ding, Dinghui Wang, Shoutao Zhang

**Affiliations:** 1 School of Electronic Engineering, Nanjing Xiaozhuang University, Nanjing 211171, China; 2 National Laboratory of Solid State Microstructures & Collaborative Innovation Center of Advanced Microstructures, School of Physics, Nanjing University, Nanjing 210093, China; 3 Jiangsu Physical Science Research Center, Nanjing 210093, China; 4 School of Materials Science and Physics, China University of Mining and Technology, Xuzhou 221116, China; 5 State Key Laboratory of Integrated Optoelectronics and Key Laboratory of UV-Emitting Materials and Technology of Ministry of Education, School of Physics, Northeast Normal University, Changchun 130024, China; zhangst966@nenu.edu.cn

**Text Correction**

The original publication [1] would benefit from additional clarification regarding the literature review, the elemental nitrogen reference states used in the formation-enthalpy calculations, and the discussion of the thermodynamic stability of several Si–N compounds. These points have now been clarified. The scientific conclusions of the published article remain unchanged.

The following corrections have been made to the Introduction and Results and Discussion sections:

1. A correction has been made to Section 1, Paragraph 3, with the following content inserted before the last sentence:

Sylvain Pitié et al. predicted several (meta)stable Si_x_N_γ_ phases, including SiN, Si_3_N_4_, SiN_2_, SiN_4_, SiN_6_, and SiN_20_, and identified nitrogen-rich Si–N compounds as high-energy-density materials [27]. Similarly, Paras Patel et al. predicted metastable Cc-SiN_6_ as a promising silicon-based high-energy-density material with high stability and strong detonation performance [28]. Other studies have also explored the Si–N system [29], with early work focusing mainly on nitrogen-rich silicon nitride phases. In contrast, the present study extends the investigation to more silicon-rich compositions and to properties that have remained largely unexplored in this system.

2. A correction has been made to Section 2.1, Paragraph 1, with the following content inserted after “For reference, the diamond-structured Si was adopted”:

The definition of the nitrogen elemental reference states is indeed a critical issue in evaluating formation enthalpies under pressure, as elemental nitrogen undergoes a series of pressure-induced structural transformations and exists in different allotropic forms at different pressures. In the 0–100 GPa range, experimental studies indicate that, after the structural transition from *Pa*-3 to *P*4_1_2_1_2 molecular nitrogen, the phase can persist to pressures above 100 GPa because of the high kinetic barrier for further phase transitions.

3. A correction has been made to Section 2.1, Paragraph 2. The original sentence is: In addition to Si_3_N_4_ and SiN_2_, two new compositions, SiN_4_ and Si_6_N, are predicted to be thermodynamically stable. The corrected text is below: 

In addition to Si_3_N_4_ and SiN_2_, two additional compositions, SiN_4_ and Si_6_N, are predicted to be thermodynamically stable. Among them, Si_6_N is a newly identified composition. Although SiN_4_ has been reported previously [27], it was found to be metastable when evaluated against theoretical elemental reference states. By contrast, using experimentally established elemental reference phases, the present calculations indicate that SiN_4_ is thermodynamically stable.

**References**

A correction has been made to Section 1, Paragraph 3, where three new references have been added.

27. Pitie, S.; Wang, B.; Larhlimi, R.; Villegas Bello, O.; Shahsavari, R.; Wu, S.; Guégan, F.; Frapper, G. Stoichiometric and Structural Diversity in the Silicon/Nitrogen Phase Diagram at Atmospheric and High Pressures. *J. Phys. Chem. C* **2025**, *129*, 7182–7195.
28. Patel, P.; Patel, S.; Dalsaniya, M.H.; Kurzydłowski, D.; Kurzydłowski, K.J.; Jha, P.K. Exploration of Si–N compounds as high energy density materials. *Phys. Chem. Chem. Phys.* **2024**, 26, 25089–25097.
29. Larhlimi, R. Prediction of Polynitrogen Materials AxNy Under Pressure (A = Sn, Si, and Bi) Using Evolutionary Algorithm and DFT Calculations. Ph.D. Thesis, Poitiers University, Poitiers, France, 2021.

With this correction, the order of some references has been adjusted accordingly. 

The authors state that the scientific conclusions are unaffected. This correction was approved by the Academic Editor. The original publication has also been updated.
