# Supplementary material for: Structural, Mechanical, and Electronic Properties of High-Hardness Silicon Tetranitride
Source: Molecules. 2025 Nov 11;30(22):4357. doi: 10.3390/molecules30224357 (PMC12654970; doi:10.3390/molecules30224357)
Supplement: Supplementary file 1 [file molecules-30-04357-s001.zip › molecules-3959207-supplementary.pdf]

Supporting Information

Structural, Mechanical, and Electronic Properties of High-Hardness Silicon Tetranitride

Lulu Liu [1,2,3,]*, Jiacheng Qi [1], Chi Ding [2], Dinghui Wang [4], and Shoutao Zhang [5]

1 School of Electronic Engineering, Nanjing Xiaozhuang University, Nanjing 211171, China
2 National Laboratory of Solid State Microstructures & Collaborative Innovation Center of Advanced Microstructures, School of Physics, Nanjing University, Nanjing 210093, China
3 Jiangsu Physical Science Research Center, Nanjing 210093, China
4 School of Materials Science and Physics, China University of Mining and Technology, Xuzhou 221116, China
5 State Key Laboratory of Integrated Optoelectronics and Key Laboratory of UV-Emitting Materials and Technology of Ministry of Education, School of Physics, Northeast Normal University, Changchun 130024, China; zhangst966@nenu.edu.cn
* Correspondence: liululu@nju.edu.cn

Index **Page**

1. Computational details··············S3

2. Enthalpy differences of *R*-3*c* SiN_4··············S4

3.Variation of the lattice constants of *R*-3*c* SiN_4··············S4

4. Electronic band structure of *R*-3*c* SiN_4 at 100 GPa··············S5

5. The -PCOHP of *P*-1 SiN_4··············S5

6. Electronic band structure of *P*-1 SiN_4··············S5

7. Phonon spectra of *P*-1 SiN_4··············S6

8. Molecular dynamics simulations of the *R*-3*c* SiN_4··············S6

9. Pair distribution functions of *P*-1 SiN_4 ··············S6

10. Property comparison of *R*-3*c* SiN_4 and *P*-1 SiN_4 phases··············S7

11. Calculated Bader charge of *P*-1 SiN_4··············S7

12. Parameters involved in calculating *R*-3*c* SiN_4 explosion performance··············S8

Computational details

Our structural prediction approach is based on a global minimization of free energy surfaces merging *ab initio* total-energy calculations with CALYPSO (Crystal structure AnaLYsis by Particle Swarm Optimization) methodology as implemented in the CALYPSO code [27-28]. The structures of stoichiometry Si_xN_y were searched with simulation cell sizes up to 4 formula units (f.u.) at 0 K and the considered pressures. In the first step, random structures with certain symmetry are constructed in which atomic coordinates are generated by the crystallographic symmetry operations. Local optimizations using the VASP code [59] were done with the conjugate gradients method and stopped when enthalpy changes became smaller than 1×10^{-5} eV per cell. After processing the first-generation structures, 60% of them with lower Gibbs free energies are selected to construct the next generation structures by PSO (Particle Swarm Optimization). 40% of the structures in the new generation are randomly generated. A structure fingerprinting technique of bond characterization matrix is applied to the generated structures, so that identical structures are strictly forbidden. These procedures significantly enhance the diversity of the structures, which is crucial for structural global search efficiency [53-56]. In most cases, structural searching simulations for each calculation were stopped after generating 1000 ~ 1200 structures (e.g., about 20 ~ 30 generations).

Supporting Figure

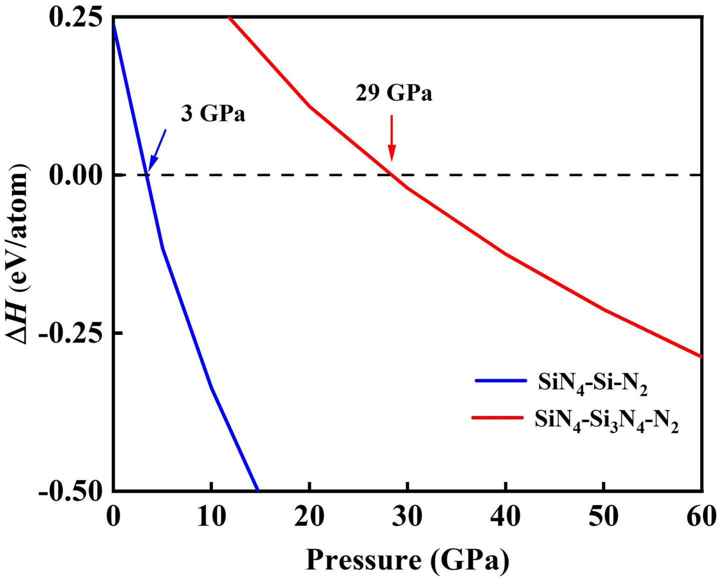

Figure S1. Calculated enthalpy differences of *R*-3*c* SiN_4 relative to the mixtures of Si_3N_4, Si, and pure nitrogen.

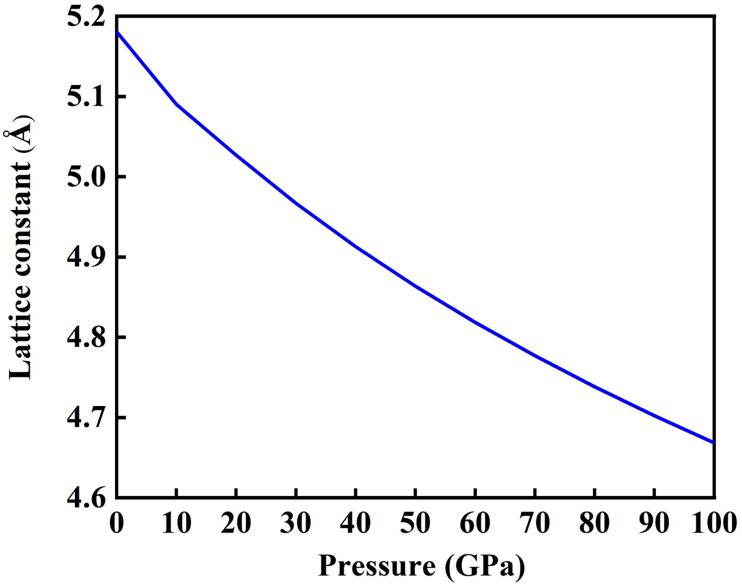

Figure S2. Variation of the lattice constants (a = b = c) of *R*-3*c* SiN_4 the unit cell with pressure.

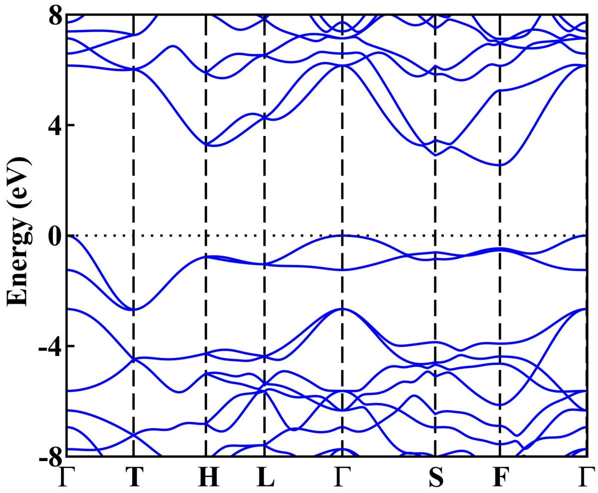

Fig S3. (a) Electronic band structure *R*-3*c* SiN_4 at 100 GPa.

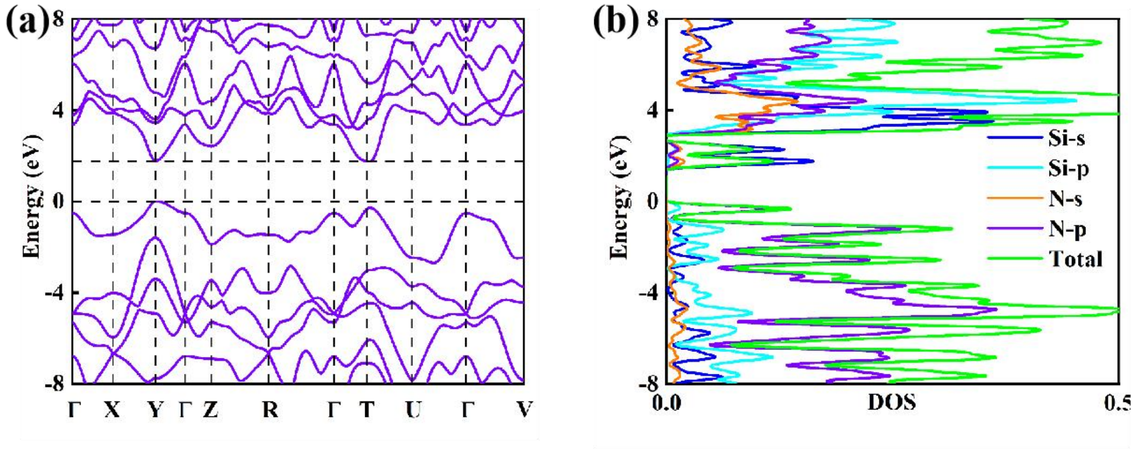

Fig S4. (a) Electronic band structure and (b) dos of *P*-1 SiN_4 at 0 GPa.

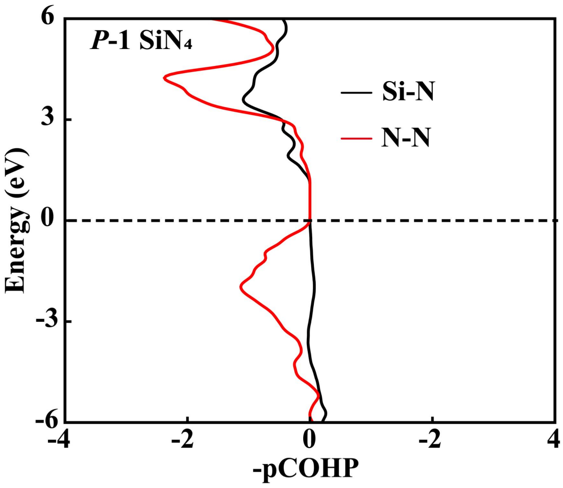

Fig S5. The -PCOHP of *P*-1 SiN_4 at 0 GPa.

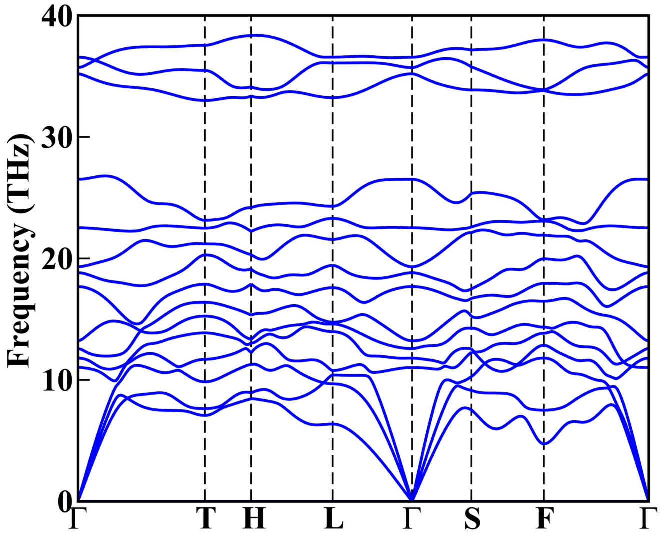

Fig S6. Phonon spectra of *P*-1 SiN_4 at 0 GPa

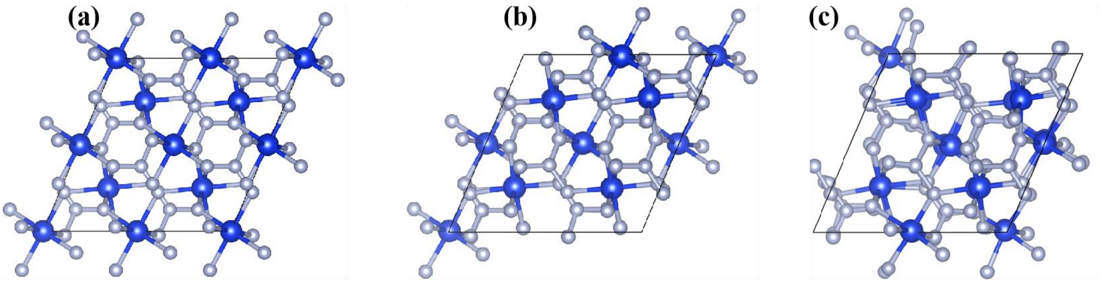

Fig S7. (a)The structure after initial optimization, (b) final structure at 300 K, and (c) final structure at 1000 K of *R*-3*c* SiN_4.

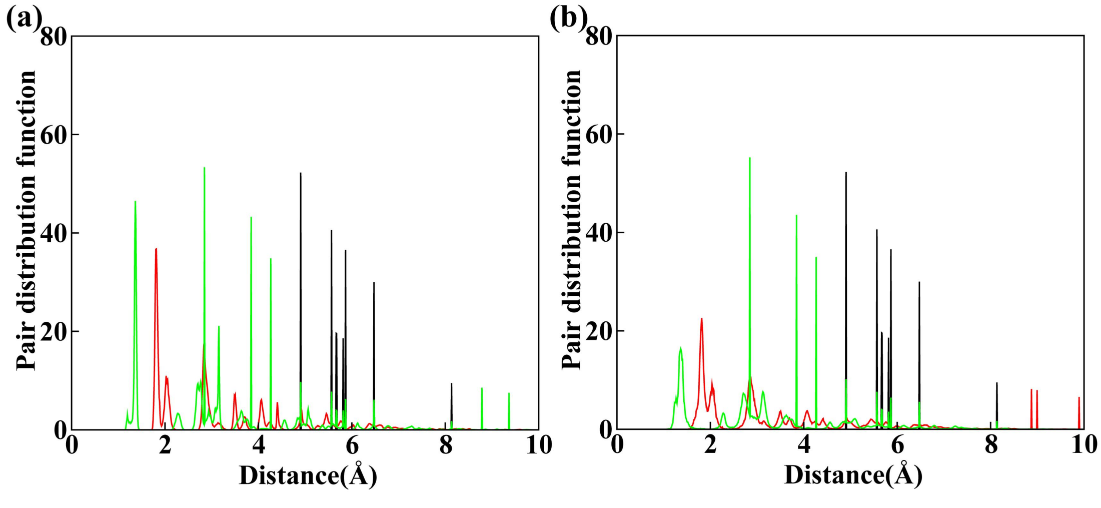

Fig S8. Pair distribution functions of *P*-1 SiN_4 at 0 GPa at 300 K (a) and 1000 K (b), respectively.

Table S1. Comparison of mechanical, electronic, and bonding properties of *R*-3*c* and *P*-1 SiN_4 phases.

**Property**	***R*-3*c* SiN_4**	***P*-1 SiN_4**
Bulk modulus (GPa)	259.53	227.38
shear modulus (GPa)	204.23	139.45
Young's modulus (GPa)	485.38	347.35
Poisson's ratio	0.18	0.25
Density (g cm^{-3})	1.86	1.66
Hardness (GPa)	31	17
Band gap (eV)	2.5	1.4
ICOHP (N-N, eV $pair^{-1}$)	-14.13	-14.83
ICOHP (Si-N, eV $pair^{-1}$)	-5.59	-5.96
Madelung Energy (Mulliken, eV $atom^{-1}$)	-4.10	-4.18
Madelung Energy (Loewdin, eV $atom^{-1}$)	-3.09	-2.67

Table S2. Charge transfer in *P*-1 SiN_4 at 0 pressure.

**Pressure (GPa)**	**Charge (|e|)**
Si	3.04
N1	-0.32
N2	-1.20
N3	-1.20
N4	-0.32
Total	0

Table S3. Calculated energy density (E_d, kJ·g^{-1}), detonation pressure (P_d, kbar), and detonation velocity (V_d, km·s^{-1}) of *R*-3*c* SiN_4. For comparison, the table also lists the explosion parameters of our calculated TNT and experimental TNT, where the superscript *expt* represents the experimental data.

	E_d	P_d	V_d
***R*-3*c* SiN_4**	1.1	228	7.11
**TNT**	3.9	189	6.76
**TNT^{expt}**	4.3	190	6.90
